# Clinical Significance of Therapeutic Drug Level Monitoring for Mycophenolate in Patients With Extrarenal Systemic Lupus Erythematosus—A Systematic Review and Meta‐Analysis

**DOI:** 10.1002/acr.80035

**Published:** 2026-04-22

**Authors:** Zahraa Qamhieh, Dalia Sriwi, Callie Saric, Tripti Singh, Christie M. Bartels, Shivani Garg

**Affiliations:** ^1^ University of Wisconsin Madison; ^2^ Yale University New Haven Connecticut

## Abstract

**Objective:**

Clinical response to mycophenolic acid (MPA) is highly heterogeneous; thus, therapeutic drug level monitoring (TDM) may help improve treatment efficacy. This systematic review and meta‐analysis examined therapeutic ranges for MPA levels associated with better outcomes and safety in patients with systemic lupus erythematosus (SLE), particularly those with extrarenal manifestations.

**Methods:**

We performed a comprehensive search of studies evaluating associations between MPA levels and clinical SLE response. Using forest plots, we calculated pooled odds of clinical response by MPA levels and measured the weighted mean differences across outcomes. Analysis was performed in all patients with SLE and separately in patients with extrarenal manifestations.

**Results:**

Among 459 reviewed abstracts, 24 met inclusion. Summarized evidence supported that clinical response was observed at MPA area under the curve at 0 to 12 hours (AUC_0–12_) ≥30 to 35 mg hr/L or trough concentration (C_trough_) ≥1.5 mg/L. At these thresholds, therapeutic MPA levels were associated with 12‐fold higher odds (95% confidence interval [CI] 5.44–27.35; *P* < 0.0001; I^2^ = 41%) of overall clinical SLE response and 15‐fold higher odds (95% CI 4.74–46.89; *P* < 0.0001; I^2^ = 61%) of response in patients with extrarenal manifestations. Additionally, MPA levels were 32 units higher (95% CIs 17.35–45.67) in overall responders with SLE and 39 units (95% CI 15.05–62.53) higher in patients with extrarenal manifestations. Although pooled analysis did not show a significant increase in adverse events, individual studies suggested safety concerns at MPA AUC_0–12_ >60 mg hr/L or C_trough_ ≥2.5 to 3 mg/L

**Conclusion:**

This study highlights the clinical utility of TDM to guide MPA dosing to balance efficacy versus safety in all patients with SLE, including those with extrarenal manifestations.

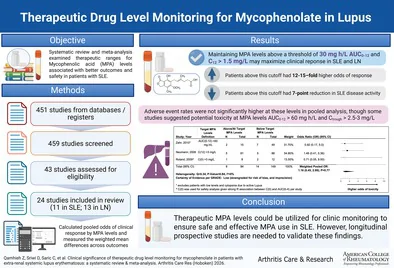

## INTRODUCTION

Systemic lupus erythematosus (SLE) is a life‐threatening autoimmune disease that disproportionately affects young female individuals and is a leading cause of premature death in young women.^1^, ^2^ Therefore, precise treatment is crucial to prolong disease‐free and damage‐free survival in all patients.^3^ As the SLE treatment armamentarium continues to expand, mycophenolic acid (MPA) remains a key therapeutic for both renal and extrarenal manifestations of SLE.^4^, ^5^ However, studies have shown a significant heterogeneity in treatment response with fixed MPA dosing, resulting in variability in SLE outcomes and adverse events.^6^, ^7^ Such heterogeneity in treatment response to MPA may be explained by a myriad of clinical factors. These include dose, drug absorption, metabolism, clearance, interactions, genetics, specific disease manifestations, and treatment adherence at individual patient level.^8^ Therapeutic drug level monitoring (TDM) of MPA could circumvent this dilemma by bypassing clinical variables. This will help optimize MPA dosing per individual patient factors to balance safety and efficacy.^9^, ^10^

**SIGNIFICANCE AND INNOVATIONS**

- This systemic review and meta‐analysis suggest the potential clinical significance of maintaining mycophenolic acid (MPA) levels above a threshold of 30 mg hr/L area under the curve at 0 to 12 hours (AUC_0–12_) and concentration at 12 hours (C_12_) >1.5 mg/L to maximize clinical response in systemic lupus erythematosus (SLE) and lupus nephritis (LN), as patients above this cutoff demonstrated 12‐ to 15‐fold higher odds of response, including in those with extrarenal manifestations.
- Additionally, patients with MPA levels above 30 mg hr/L AUC_0–12_ and C_12_ >1.5 mg/L had a significant seven‐point reduction in SLE disease activity.
- Adverse event rates were not significantly higher at these levels in pooled analysis, though some individual studies suggested potential toxicity at MPA levels AUC_0–12_ >60 mg hr/L and trough concentration >2.5 to 3 mg/L.
- Therapeutic MPA levels could be used for clinic monitoring to ensure safe and effective MPA use in SLE. However, longitudinal prospective studies are needed to validate these findings and establish a roadmap to personalize dosing in SLE and LN, balancing safety and efficacy.

TDM has been successful in renal transplant and lupus nephritis (LN) to guide safe and effective dosing of tacrolimus.^11^, ^12^ A similar strategy could be leveraged for MPA in SLE, in which accurate estimates of MPA plasma concentration could guide optimal MPA dosing. However, TDM of MPA is not routinely used during SLE visits because (1) it is unclear how MPA levels should be routinely measured during visits (eg, area under the curve [AUC] vs trough concentration [C_trough_] vs peak concentration), (2) thresholds or therapeutic ranges for MPA levels that are associated with better SLE outcomes versus lower adverse events are largely unknown, and (3) limited data are available regarding clinical variables that might influence MPA plasma concentrations. A recent meta‐analysis shed new evidence to address existing gaps in TDM for MPA in patients with LN, but results were limited to renal outcomes.^13^ Given that MPA is a key medication for moderate–severe extrarenal SLE manifestations,^14^ similar data on TDM for extrarenal manifestations are needed. Objectives of this meta‐analysis and systemic review were to (1) examine therapeutic thresholds for MPA levels that are associated with better outcomes and safety in all patients with SLE, and particularly those with extrarenal SLE manifestations, (2) identify clinical variables that affect MPA plasma concentrations to inform dosing and enhanced monitoring in those subgroups, and (3) clarify reliable methods that can be used to measure MPA levels in clinics.

## MATERIALS AND METHODS

A comprehensive literature search was performed using Medical Subject Headings (MeSH) headings and keywords in the Medline, Embase, CINAHL, clinicaltrials.gov, and Web of Science databases with references reviewed. Results were limited to articles published between January 1, 1990, and April 30, 2024. The search included the MeSH and keywords “systemic lupus erythematosus” OR “lupus” OR “SLE” OR “lupus nephritis” OR “lupus vasculitis” OR “CNS lupus” OR “discoid lupus” OR “cutaneous lupus” AND “concentrations” OR “weight” OR “measures” OR “levels” OR “quantification” AND “mycophenolate” OR “mycophenol” OR “Cellcept” OR “MMF.” Additional studies were identified through checking the references of articles included for full text review using Medline, Web of Science, and CINAHL. Finally, we manually searched the references and citations in reviews, meta‐analysis, expert opinions, and articles selected for full text review until we achieved saturation in results and no new studies were identified. We did not filter studies by language. Study screening was performed using Covidence, after which included studies were organized into an Excel sheet for data abstraction.

During the initial screening, two independent reviewers (DS and CS) screened the retrieved articles by reading the titles and abstracts to identify the studies that met the a priori list of inclusion criteria (Supplementary Table 1). Any discrepancy in the decision to include a study was resolved by an independent reviewer (SG). We included observational and interventional studies on human participants, measuring an association between MPA levels and clinical response (efficacy or safety) in patients with SLE with extrarenal or renal manifestations. Both mycophenolate mofetil (MMF) and MPA were considered. Case reports, expert opinions, narrative reviews, and conference abstracts were excluded. The Preferred Reporting Items for Systematic Reviews and Meta‐Analysis guidelines and checklist were used for the literature identification process^15^ (Supplementary Figure 1). A detailed protocol can be found on PROSPERO (CRD42024525955). Quality assessment of observational studies used the Newcastle–Ottawa scale (good: 7–9; fair: 4–6; poor: 0–3).^16^ The Cochrane Collaboration Risk Assessment tool was used for interventional studies.^17^ Certainty of evidence for each outcome was evaluated using the Grading of Recommendations Assessment, Development, and Evaluation (GRADE) framework.^18^ An overall score was generated using the standard guidelines on quality assessment from the Agency of Healthcare Research and Quality and the Cochrane handbook.^19^ Two reviewers (DS and CS) independently performed quality assessments, and discrepancies were resolved by the third reviewer (SG).

A data extraction form was developed and pilot tested by the lead investigator (SG). Data abstraction form was then used by two independent reviewers (DS and CS) to abstract an a priori list of variables including patient demographics, disease characteristics, mycophenolate dose, concomitant SLE therapies, disease activity, response criteria, response rate, other clinical variables (eg, estimated glomerular filtration rate [eGFR], albumin), MPA levels and measurement method, adverse events (toxicity), and other relevant outcomes. When appropriate, authors of the studies were contacted for additional information. Disagreements were settled by two reviewers (ZQ and SG). Studies comparing clinical response in SLE manifestations with higher or therapeutic mycophenolate levels versus a comparator group were included in the meta‐analysis. If no comparator group was present, then the findings were systematically reviewed, and narrative synthesis was performed. Given the study design and use of published de‐identified aggregate data for pooled analysis, this study was exempted for a review by the University of Wisconsin, Madison, Institutional Review Board office.

### Research question

Our research question, structured using the population, intervention, comparator, outcome (PI[E]CO) framework, focused on patients with SLE, including both extrarenal and renal manifestations. We examined whether higher or therapeutic MPA levels, compared with lower or subtherapeutic levels, were associated with clinical response, disease activity scores, adverse events, and variability in MPA concentrations.

### Primary objective: identify therapeutic thresholds for MPA level monitoring in SLE

The primary objective of this systematic review and meta‐analysis was to define therapeutic thresholds for MPA levels that are associated with safe and effective MPA use in patients with SLE. We examined the following:

1. associations between MPA levels and clinical response in patients with extrarenal SLE and in all patients with SLE (renal and extrarenal),
2. MPA levels and adverse event occurrence in patients with extrarenal SLE and in all patients with SLE including both extrarenal and renal SLE outcomes.

### Secondary objectives: clinical variables that affect MPA levels

To understand how clinical variables impact MPA plasma concentrations and how MPA levels can be measured or performed during clinic visits, we did the following:

1. examined associations among clinical variables such as ethnicity, eGFR, albumin, MPA dose, other medicines, and MPA plasma concentrations,
2. summarized data on different methods that were used to measure MPA levels and the potential clinical utility of each method to inform TDM intervention for routine visits.

### Statistical analysis

We examined the pooled odds of clinical response in patients with extrarenal SLE manifestations and overall SLE (renal and extrarenal) by high or therapeutic MPA levels versus low or subtherapeutic MPA levels. We defined clinical response in patients with extrarenal SLE manifestations per primary studies: (1) active SLE or SLE flare defined as British Isles Lupus Assessment Group A or B score,^20^ (2) active SLE defined as Systemic Lupus Erythematosus Disease Activity Index (SLEDAI),^21^ (3) markers of SLE disease activity (low complements and high double‐stranded DNA antibody), and (4) SLE response defined by Lupus Low Disease Activity State.^22^ For LN, definitions included the following: (1) Kidney Disease Improving Global Outcomes complete or partial renal remission and renal or LN flare criteria,^23^ and (2) American College of Rheumatology (ACR) complete or partial renal remission and renal or LN flare criteria.^24^ Clinical response outcomes were analyzed separately. To define safe MPA levels, we compared MPA levels in those with versus without adverse events.

For all analysis, we used forest plots to calculate the pooled odds ratios (ORs; 95% confidence interval [CI]) of clinical response in higher or therapeutic mycophenolate levels versus lower or subtherapeutic levels. Additionally, pooling data from studies reporting differences in MPA levels by clinical response, we calculated the weighted mean difference (95% CIs) in MPA levels by clinical response. Finally, we also calculated weighted mean difference (95% CIs) in SLEDAI score by MPA levels when available. A narrative synthesis was performed for studies without a comparator group or without aggregate data by clinical response or MPA levels. All analyses were completed using a random‐effects meta‐analysis model and tau2 was calculated using the restricted maximum likelihood method from the metafor package in R (version 3.4.1).^25^ This model and tau2 method account for between‐study heterogeneity by estimating a variance component for the true effect size across studies, and it appropriately weights studies based on their sample size and design characteristics. Heterogeneity was assessed using I^2^. We performed sensitivity analysis stratified by study design to assess the robustness of our findings. Publication bias was evaluated by generating funnel plots. For each objective, certainty of evidence was rated using GRADE recommendations.

## RESULTS

### Study characteristics

Our literature search yielded 451 articles plus 9 additional articles through manual search. After removing duplicates, 459 articles were included in the initial screening (Supplementary Figure 1). Next, 42 studies met the priori inclusion criteria to determine eligibility for a full text review (Supplementary Table 1). Ultimately, 24 studies were selected for final date extraction and quality assessment,^6^, ^26^, ^27^, ^28^, ^29^, ^30^, ^31^, ^32^, ^33^, ^34^, ^35^, ^36^, ^37^, ^38^, ^39^, ^40^, ^41^, ^42^, ^43^, ^44^, ^45^, ^46^, ^47^, ^48^ prospective cohort (n = 12),^6^, ^28^, ^31^, ^33^, ^35^, ^36^, ^37^, ^39^, ^40^, ^45^, ^47^ retrospective cohort (n = 10),^26^, ^27^, ^29^, ^30^, ^32^, ^34^, ^42^, ^43^, ^46^, ^48^ clinical trial (n = 1),^38^ and cross‐sectional (n = 1).^41^ To avoid duplication, we did not include data abstracted from prior systematic reviews. Instead, we reviewed the original primary studies directly and extracted data from these articles.^13^ A total of 24 observational studies were single center, 9 were completed in Europe,^6^, ^26^, ^30^, ^31^, ^34^, ^36^, ^39^, ^44^, ^47^ 13 were completed in Asia,^27^, ^28^, ^29^, ^32^, ^33^, ^35^, ^37^, ^38^, ^40^, ^41^, ^42^, ^43^, ^45^, ^46^, ^48^ and all were conducted after 2008 (Table 1). The overall Newcastle–Ottawa scale for risk of bias assessment showed 16 “good,“^6,26,28,29,31,32,35,37–39,41–43,45–48^ 4 “fair,“^27^, ^33^, ^34^, ^44^ and 3 “poor”^30^, ^36^, ^40^ (Table 1).

**Table 1:** Study and patient demographics*

Author (year)	Study population	LN and/or SLE	MPA level threshold	Outcomes observed by MPA levels	Bias
Neumann et al (2008)^31^	N = 13; mean age 39.6 years; F = 92%; site: Austria	LN 38%; SLE 68%	C_12_ 3.5–4.5 mg/L	Active SLE defined as BILAG A/B^a^ AEs: infection, anemia, GI upset	NOS: good
Fujinaga et al (2008)^40^	N = 4; mean age 11.75 years; F = 75%; site: Japan	LN 100%	C_0_ 2–5 mcg/mL	LN flare defined as ↑ in UPCR to >1 or ↑ in Cr by 50%	NOS: poor
Roland et al (2009)^36^ ^b^	N = 20; mean age 41 ± 15 years; F = 65%; wt. 69.5 ± 13.4 kg; site: France	LN 50%; SLE 50%	AUC_0–4_ 28.4 ± 13.6 mg hr/L or C_0_ 3.1 ± 2.2 mg/L	SLE activity defined as ↓ complement and ↑ dsDNA levels AEs: GI upset, neutropenia Relevant clinical factors: albumin, wt., CrCl	NOS: poor
Zahr et al (2010)^6^ ^b^	N = 71; mean age 35 years, F = 86%; BMI 24.3; site: France	LN 84%; SLE 16%	AUC_0–12_ ≥ 35 μg h/mL	Active SLE defined as SLEDAI ≥6 and/or BILAG A/B^a^ AE: cytopenia Relevant clinical factors: albumin, CrCl, MMF dose	NOS: good
Djabarouti et al (2010)^47^	N = 26; mean age 46 years; F = 61%; wt. 60 kg; 100% White; site: France	SLE 100%	C_12_ ≥ 3 mg/L	SLE flare defined as ↑in SLEDAI by at least three points in six months Relevant clinical factors: albumin, GFR, steroids	NOS: good
Lertdumrongluk et al (2010)^35^, ^b^	N = 18; mean age 33 years; F = 89%; BMI 22.2; site: Thailand	LN 100%	AUC_0–12_ > 45 mg hr/L	LN response per ACR PR definition^c^ AEs: GI upset, cytopenia, infection	NOS: good
Sagcal‐Gironella et al (2011)^33^, ^b^	N = 19; mean age 16.9 years; F = 95%; wt. 66.6 kg; AA = 58%; site: United States	LN 84%; SLE 16%	AUC_0–12_ ≥ 30 mg hr/L	SLE disease activity defined as BILAG score reduction^d^	NOS: fair
Daleboudt et al (2012)^34^, ^b^	N = 16; mean age 33.2 ± 12.1 years; F = 94%; wt. 67 ± 11.5 kg; 75% White; site: the Netherlands	LN 100%	AUC_0–12_ 60–90 mg hr/L	LN remission per KDIGO guidelines^e^ AEs: GI upset, infections	NOS: fair
Kittanamongkolchai et al (2013)^38^, ^b^	N = 19; mean age 30.5 years; F = 95%; BMI 21.2; 100% Asian; site: Thailand	LN 100%	C_1_ >13 mg/L or AUC_0–12_ > 45 mg hr/L	LN response per ACR definition^c^ AEs: GI upset, infection	Cochrane: low
Woillard et al (2014)^30^	N = 36; mean age 12.0 years; F = 72%; BMI 20.72; site: France	SLE 100%	AUC_0–12_ > 44 mg l^−1^ hours	Active SLE defined as SLEDAI ≥6 Relevant clinical factor: albumin	NOS: poor
Streicher et al (2014)^39^	N = 23; mean age 38 years; F = 78%; site: France	SLE 100%	C_0_ ≥ 2.5–3 mg I^−1^	SLE flare defined as ↑in SLEDAI by at least three points in 12 months AE: GI upset	NOS: good
Alexander et al (2014)^37^, ^b^	N = 34; mean age 25 vs 31.1 years; F = 100% vs 84%; 33.5% Asian; site: India	LN 100%	AUC_0–12_ ≥ 30 mg hr/L	LN remission per CR KDIGO guidelines^d^ AEs: infection, cytopenia Clinical factors: albumin, wt.	NOS: good
Zabotti et al (2014)^44^, ^b^	N = 5; mean age 34.2 years; F = 80%; site: Italy	LN 100%	AUC_0–12_ 45–60 mg hr/L	LN remission per KDIGO guidelines^e^ AEs: GI upset, cytopenia	NOS: fair
Abd Rahman et al (2015)^45^, ^b^	N = 25; median age 32.6 years; F = 80%; wt. 61.1 kg; site: Malaysia	LN 100%	AUC_0–12_ > 30 mg^−1^	LN response per ACR PR definition^c^ AEs: GI upset, infection, cytopenia	NOS: good
Hui‐Yuen et al (2016)^29^, ^b^	N = 17; mean age pediatric; F = 63%; 47.1% Black; site: United States	LN 100%	G_6_ 35–100 μg/mL or MPA C_6_ 1–5 μg/mL	LN remission at three months defined as proteinuria <500 mg/24 hr	NOS: good
Yap et al (2020)^28^, ^b^	N = 88; mean age 43.8 years; F = 88%; 100% Asian; site: China	LN 100%	C_12_ > 1.5 mg/L	LN flare defined as ↑in proteinuria ≥1 g/day and/or ↑ in Cr by ≥15% AEs: infections, cytopenia	NOS: good
Katsuno et al (2018)^32^, ^b^	N = 20; mean age 39.5 years; F = 90%; BMI 19.6; 100% Asian; site: Japan	LN 100%	N/A	LN remission per CR KDIGO guidelines^e^ AE: infections	NOS: good
Godron‐Dubrasquet et al (2020)^26^, ^b^	N = 27; mean age 12.4 years; F = 89%; site: France	LN 100%	AUC_0–3_ > 45 mg hr/L	LN response per KDIGO guidelines^e^ AEs: GI upset, infections, cytopenia clinical factors: albumin, wt.	NOS: good
Chen et al (2020)^43^	N = 67; mean age 12–13 years; F = 96% vs 76%; wt. 45 years; site: China	SLE 100%	AUC_0–12_ > 50 μg hr/mL; C_trough_ > 1.7 μg/mL	SLE activity defined as SLEDAI ≥ 6 Relevant clinical factors: albumin, steroids	NOS: good
Ye et al (2021)^27^	N = 107; mean age 133.83 months; F = 76%; BMI 20.78; site: China	LN 81%; SLE 18%	AUC_0–24_ 100.39 mg hr/L or AUC_0–12_ 50.20 mg hr/L	SLE activity measured per SLEDAI score Correlation of SLE comorbidities (AKI, PNA, DM) by MPA level	NOS: fair
Abe et al (2021)^48^	N = 119; mean age 38 years; F = 85%; wt. 55 kg; 100% Asian; site: Japan	LN 59%; SLE 41%	N/A	SLE response defined as LLDAS^f^ AE: GI upset, infection, cytopenia	NOS: good
Chariyavilaskul et al (2022)^46^, ^b^	N = 19; median age 29 years; F = 100%; site: Thailand	LN 100%	C_0.5_ ≥ 2.03 μg/mL or AUC_0–12_ ≥ 45 μg hr/mL	LN response per KDIGO guidelines^e^ Correlation b/w MPA AUC and trough levels Relevant clinical factor: albumin	NOS: good
Navamani et al (2022)	N = 25; mean age 15 years; F = 80%; wt. 48.6 kg; 100% Asian; site: India	LN 100%	N/A	Active SLE defined by SLEDAI ≥6 Relevant clinical factor: albumin	NOS: good
Mizaki et al (2023)^42^	N = 34; mean age 39 years; F = 82%; wt. 54.7 kg; 100% Asian; site: Japan	LN 100%	AUC_0–12_ 30–45 μg hr/mL; C_max_ < 24.4 μg/mL	No clinical outcomes reported AEs: GI upset, infection	NOS: good

* AA, African American; ACR, American College of Rheumatology; AE, adverse event; AKI, acute kidney injury; AUC_0–4_, area under the curve at zero to four hours; BILAG, British Isles Lupus Assessment Group; BMI, body mass index; b/w, between; C_12_, concentration at 12 hours; C_max_, maximum mycophenolic acid concentration among serial measurements; CR, complete remission; Cr, creatinine; CrCl, creatinine clearance; DM, diabetes mellitus; dsDNA, double‐stranded DNA; eGFR, estimated glomerular filtration rate; G_6_, mycophenolic acid glucuronide 6 hours levels; GI, gastrointestinal; KDIGO, Kidney Disease Improving Global Outcomes; LLDAS, Lupus Low Disease Activity State; LN, lupus nephritis; MMF, mycophenolate mofetil; MPA, mycophenolic acid; N/A, not applicable; NOS, Newcastle–Ottawa scale; PNA, pneumonia; PR, partial remission; SLE, systemic lupus erythematosus; SLEDAI, Systemic Lupus Erythematosus Disease Activity Index; UPCR, urine protein Cr ratio; wt., weight.

^a^ BILAG A/B: severe to moderate disease activity in at least one organ system.

^b^ These studies were included in the Wuttiputhanun et al^13^ meta‐analysis.

^c^ ACR CR: normal or ≤25% decline of GFR from baseline; UPCR <0.2; inactive urinary sediment. PR: normal or increased eGFR by 25% if the baseline was abnormal; 50% reduction of 24‐hour urine protein <2 g/day; inactive urinary sediment.

^d^ Reduction in mean time adjusted BILAG score (in percent) = (MTA‐BILAGPost‐MMF/MTA‐BILAGPre‐MMF) × 100%. Pre‐MMF was measured up to six months before initiation of MMF; Post‐MMF was measured starting two weeks after initiation of MMF.

^e^ KDIGO CR: proteinuria below 0.5 g/day and stable serum creatinine levels or less than 25% higher than at the start of treatment; PR: more than 50% reduction in proteinuria and no increase in serum creatinine levels; flare: not reaching the criteria for partial response.

^f^ LLDAS: SLEDAI ≤4, no high steroid use, no major organ involvement, stable or improving clinical condition, absence of flare or new symptoms.

### Study population and disease characteristics

Patient demographics per individual studies are summarized in Table 1. Approximately 940 patients with SLE were included. The mean age across studies ranged from 11.7 years (pediatric cohort) to 47 years (adult cohort). The proportion of female participants ranged from 61% to 100%. Among included studies, extrarenal SLE outcomes were assessed in 11 studies^6^, ^27^, ^30^, ^31^, ^33^, ^36^, ^39^, ^41^, ^43^, ^47^, ^48^ (including 1 study that used serological parameters to define active SLE^36^), whereas 13 studies examined renal outcomes.^26,28,29,32,34,35,37,38,40,42,44–46^

### Association between MPA levels and extrarenal SLE outcomes

**Figure 1:**
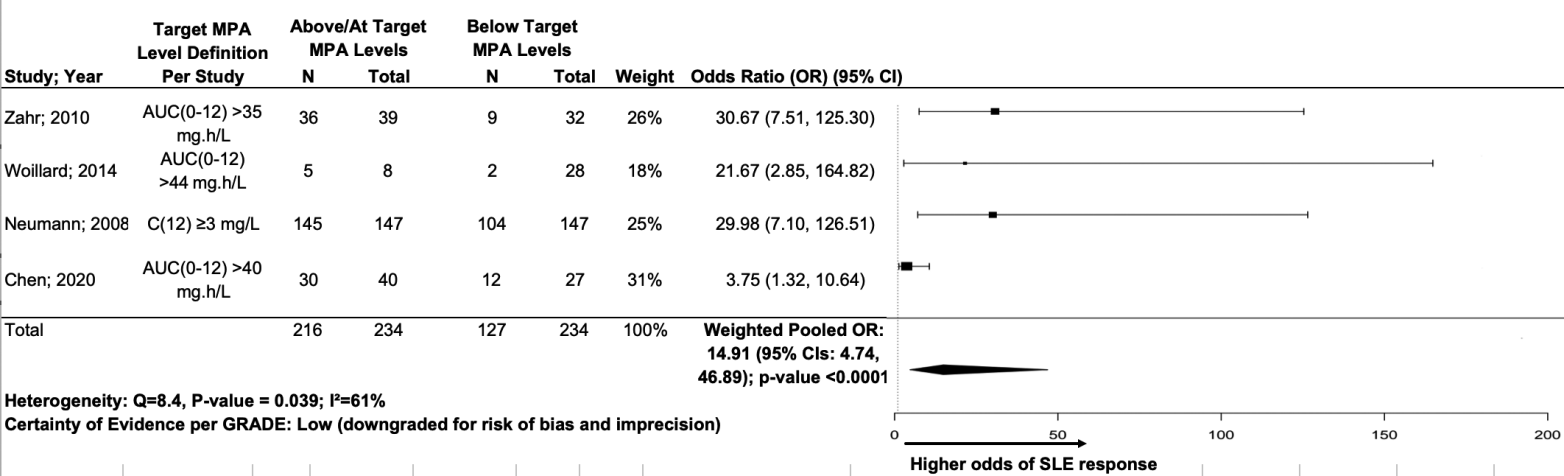
Forest plot showing the odds of clinical response in patients with systemic lupus erythematosus (SLE) with extrarenal manifestations by target mycophenolic acid (MPA) levels. AUC(0–12), area under the curve at 0 to 12 hours; CI, confidence interval; GRADE, Grading of Recommendations Assessment, Development, and Evaluation; OR, odds ratio.

**Figure 2:**
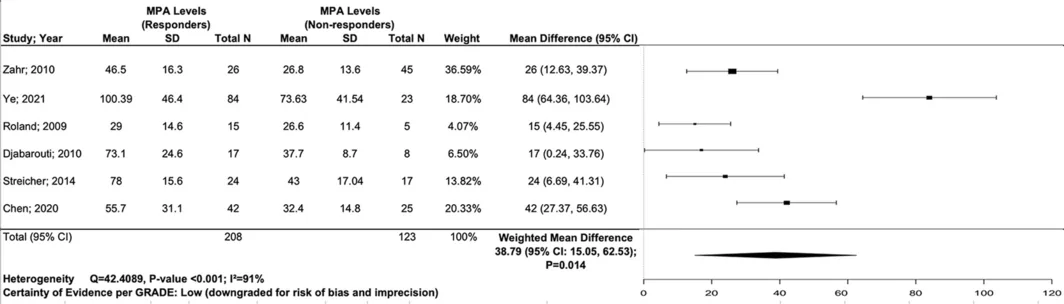
Forest plot showing the weighted mean difference in mycophenolic acid (MPA) levels by clinical response in patients with systemic lupus erythematosus with extrarenal manifestations. CI, confidence interval; GRADE, Grading of Recommendations Assessment, Development, and Evaluation.

**Figure 3:**
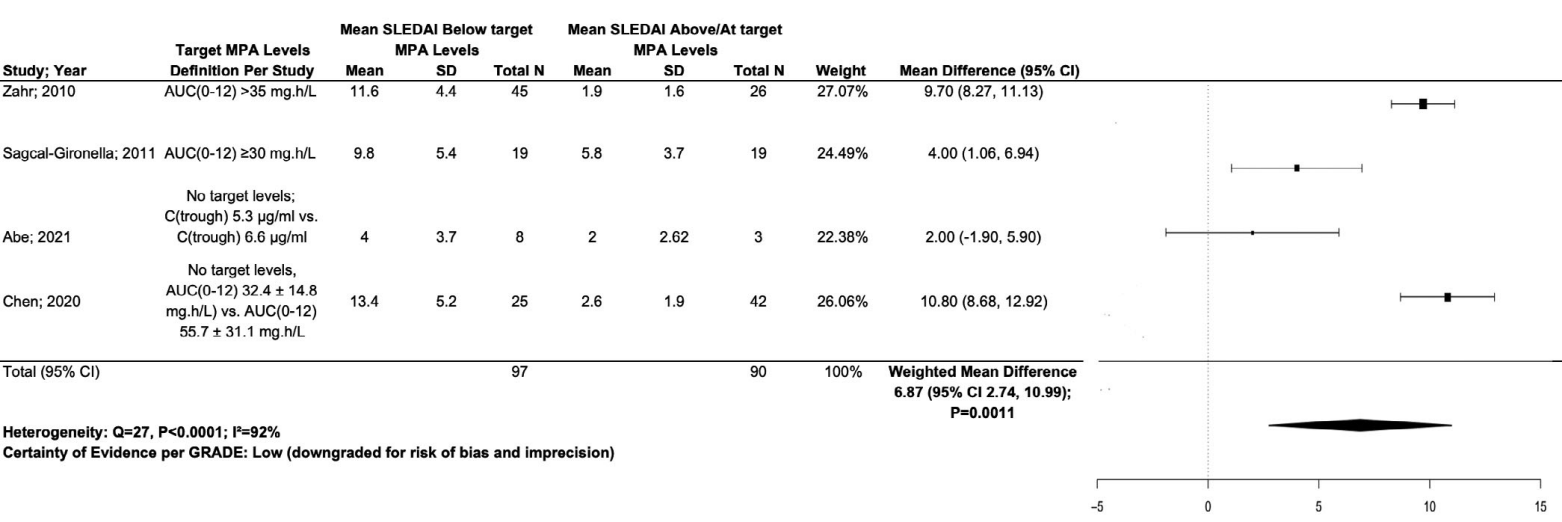
Forest plot showing the weighted mean difference in systemic lupus erythematosus (SLE) disease activity by higher or therapeutic mycophenolic acid (MPA) levels in patients with SLE with extrarenal manifestations. AUC(0–12), area under the curve at 0 to 12 hours; CI, confidence interval; C(trough), trough concentration; GRADE, Grading of Recommendations Assessment, Development, and Evaluation; SLEDAI, Systemic Lupus Erythematosus Disease Activity Index.

A total of 10 studies examined association between MPA levels and extrarenal SLE outcomes.^6^, ^27^, ^30^, ^31^, ^33^, ^36^, ^39^, ^43^, ^47^, ^48^ We performed three different pooled analyses (Table 2). First, we calculated OR (95% CI) of extrarenal SLE response by higher or therapeutic MPA levels including data from four studies (Figure 1).^6^, ^30^, ^31^, ^43^ This analysis showed 15‐fold higher pooled odds of response in groups with higher or therapeutic MPA levels (pooled OR 14.91, 95% CI 4.74–46.89; *P* < 0.0001; I^2^ = 61%; Figure 1). Next, we calculated weighted mean difference (95% CI) in MPA levels by SLE response using data from six studies (Figure 2).^6^, ^27^, ^36^, ^39^, ^43^, ^47^ We found that MPA levels were higher in responders’ versus nonresponders’ with a weighted mean difference of 38.79 units (95% CI 15.05–62.53); *P* = 0.014; I^2^ = 91%; Figure 2). Finally, we calculated the weighted mean difference in SLE disease activity scores by higher or therapeutic MPA levels, including data from four studies that used the same SLEDAI.^6^, ^33^, ^43^, ^48^ In this analysis, SLEDAI score was seven points lower in groups with higher or therapeutic MPA levels (weighted mean difference 6.87, 95% CI 2.74–19.99; *P* = 0.0011; I^2^ = 92%; Figure 3). Certainty of evidence was rated as very low for extrarenal outcomes by GRADE, primarily due to imprecision and study limitations. One study, not included in the meta‐analysis, noted a significant difference in the mean MPA AUC at 0 to 12 hours (AUC_0–12_) levels between active versus inactive pediatric patients with SLE (69 vs 38 μg h/mL; *P* = 0.003).^41^

**Table 2:** Clinical outcomes in pts. w/ SLE w/ extrarenal and renal manifestations by MPA levels*

Author (year)	MMF dose	MPA level target	Outcomes	Conclusion
Djabarouti et al (2010)^47^, ^a^	2 g/d	C_12_ ≥ 3 mg/L	Median MPA levels in pts. w/ SLE w/ flare vs no flare (1.5 vs 3.7); *P* = 0.008 MPA C_12_ ≥3 mg/L has 92% NPV for SLE flare Albumin, GFR, and steroids do not correlate w/ MPA levels	Target MPA C_12_ ≥ 3 mg/L is associated w/ a 92% NPV for SLE flare
Woillard et al (2014)^30^, ^a^	728 mg/d	AUC_0–12_ > 44 mg l^−1^ h	21‐fold ↑ odds of active SLE w/ MPA level <44 mg l^−1^ (95% CI 2.3–196.1; *P* = 0.007) Albumin does not correlate w/ MPA levels	MPA AUC_0–12_ >44 mg l^−1^ h is associated w/ 21‐fold lower odds of SLEDAI <6
Chen et al (2020)^43^, ^a^	20–40 mg/kg BID	AUC_0–12_ > 50 μg h/mL or C_trough_ > 1.7 μg/mL	Mean MPA AUC_0–12_ in pts. w/ active vs inactive SLE (32.4 ± 14.8 vs 55.7 ± 31.1); *P* < 0.0001 MPA C_trough_ levels higher by 1.2 μg/mL in inactive SLE (*P* < 0.0001) Significant correlation b/w MPA levels and albumin; steroids	Better SLEDAI w/ target MPA AUC_0–12_ >50 μg h/mL or C_trough_ >1.7 μg/mL
Neumann et al (2008)^31^, ^a^	1 g BID	C_12_ 3.5–4.5 mg/L	At MPA levels <3 mg/L, 29% of samples were from pts. w/ active SLE All pts. maintained remission w/ MPA level ≥3.5 mg/L Mild AEs in 31% of pts. w/ MPA levels ≥4.5 mg/L	MPA C_12_ levels b/w 3.5–4.5 mg/L reduce disease activity and AEs
Sagcal‐Gironella et al (2011)^33^, ^a^	1,973 mg/d	AUC_0–12_ ≥ 30 mg hr/L	pts. w/ MPA level ≥30 mg hr/L had greater percent reduction (36%) in BILAG score; *P* = 0.002	Target MPA AUC_0–12_ ≥30 mg hr/L linked w/ ↑ SLE disease control
Navamani et al (2022)^49^, ^a^	600 mg/m^2^	N/A	Mean MPA levels in pts. w/ active vs inactive SLE (38.46 ± 14.3 vs 69 ± 19.24); *P* = 0.003 Albumin does not correlate w/ MPA levels	TDM of MPA AUC_0–12_ may help ↑ SLE disease control
Ye et al (2021)^27^, ^a^	1,000 mg/d	AUC_0–12_ 50.20 mg hr/L	Mean MPA level in pts. w/ SLE comorbidities vs not (73.63 ± 41.54 vs 100.4 ± 46.4); *P* < 0.001 ↑ MPA level is associated w/ ↓ risk of DM, AKI, PNA; OR 0.991 (95% CI 0.982–0.999); *P* = 0.046	Target MPA AUC_0–12_ 50.20 mg hr/L ↓ risk of SLE comorbidities (DM, AKI, PNA)
Abe et al (2021)^48^, ^a^	1.5–2 g/d	N/A	3 of 11 pts. achieved LLDAS w/ MPA C_trough_ 8.3, 6.6, 3.5 μg/mL LLDAS achievement rate was ↓ in pts. w/ AEs vs no AEs (35% vs 65%; *P* = 0.009)	↑ MPA C_trough_ levels increases LLDAS but increases AE risk
Streicher et al (2014)^39^, ^a^	2 g/d	C_0_ ≥2.5–3 mg I^−1^	Median MPA levels in pts. w/ SLE flare vs pts. w/o flare (1.6 vs 2.95); *P* = 0.048 MPA C_0_ ≤3 has 80% NPV for SLE flare and did not increase AE risk	Target MPA C_0_ ≥2.5–3 mg I^−1^ could reduce SLE flares
Zahr et al (2010)^6^, ^a^	1,846 mg/d	AUC_0–12_ ≥ 35 μg h/mL	Mean MPA level in pts. w/ active vs inactive SLE (26.8 ± 13.6 vs 46.5 ± 16.3); *P* < 0.0001 MPA AUC_0–12_ correlates w/ lower SLEDAI (r = −0.64; *P* < 0.0001) MPA AUC_0–12_ is associated w/ SLEDAI (OR 0.89, 95% CI 0.83–0.96) No correlation b/w MPA levels and AEs Multivariate analysis revealed significant correlation b/w MPA level and serum albumin, CrCl	Target MPA AUC_0–12_ ≥35 μg h/mL is associated w/ ↑ SLE disease control w/o ↑ in AEs
Roland et al (2009)^36^, ^b^	1,600 mg/d	AUC_0–4_ 28.4 ± 13.6 mg hr/L or C_0_ 1–5 mg/L	MPA AUC_0–4_ level in pts. w/ low vs normal C3 (26.6 ± 11.4 vs 29.0 ± 14.6); *P* = 0.760 MPA AUC_0–4_ level in pts. w/ low vs normal C4 (24.1 ± 8.2 vs 30.7 ± 5.6); *P* = 0.362 MPA C_0_ level in pts. w/ low vs normal C4 (1.7 ± 1.2 vs 3.8 ± 2.3); *P* = 0.043 No significant correlation b/w MPA C_0_ and C3, dsDNA Mild AEs in three pts. w/ MPA level 17–30. No significant correlation b/w MPA level and albumin, wt., CrCl	MPA AUC_0–4_ or C_0_ can be used for MPA TDM. Target C_0_ 1–5 mg/L is linked w/ ↑ SLE disease control
Godron‐Dubrasquet et al (2020)^26^, ^c^	1,190 mg/m^2^/day	AUC_0–3_ > 45 mg hr/L	3.6‐fold ↑ odds of LN response in pts. w/ MPA level >45 (95% CI 2.4–9.5) Higher MPA level in responders w/ LN by 20 mg hr/L; *P* < 0.01 Mild AEs in 33% of pts. w/ median level 44 No significant correlation b/w MPA level and albumin, wt.	MPA‐AUC_0–3_ >45 mg hr/L is linked w/ 3.6‐fold ↑ odds of LN remission
Yap et al (2020)^28^, ^c^	1,047 mg/d	C_12_ > 1.5 mg/L	Lower MPA levels in pts. w/ LN flare by 1.1 mg/L; *P* = 0.041 MPA levels in pts. w/ vs w/o infection (4.3 ± 2.6 vs 1.8 ± 1.2) Mean MPA levels (2.9) in 10 pts. w/ anemia vs pts. w/o anemia (1.7 ± 1.1); *P* = 0.004	MPA C_12_ >1.5 mg/L ↓ LN flare but ↑ risk of infections and anemia
Hui‐Yuen et al (2016)^29^, ^c^	600 mg/m^2^ BID	MPA C_6_ 1–5 μg/mL	Mean MPA C_6_ level in responders vs nonresponders w/ LN (3.26 ± 2.02 vs 3.02 ± 1.76) Mean MPA‐G_6_ level in responders vs nonresponders w/ LN (44.2 vs 29.88); *P* = 0.5	MPA C_6_ or G_6_ not associated w/ LN response
Katsuno et al (2018)^32^, ^c^	1,000 mg BID	N/A	Median MPA AUC_0–12_ level in responders vs nonresponders w/ LN (52.6 vs 43.5); *P* = 0.09 C_0_ levels correlate w/ MPA AUC_0–12_; R = 0.73 Infection in five pts. w/ median MPA level 51.2; *P* = 0.92	TDM of MPA AUC_0–12_ or C_0_ may allow for ↑ LN disease control
Daleboudt et al (2013)^34^, ^c^	1.9 g/d	AUC_0–12_ 60–90 mg hr/L	When maintained at MPA levels 60–90, 69% of pts. had complete LN remission and 19% had partial remission Mild AEs w/o significant correlation to MPA level in 37.5% of pts.	Therapeutic MPA levels: MPA AUC_0–12_ 60–90 mg hr/L
Alexander et al (2014)^37^, ^c^	1.5–2 g/d	AUC_0–12_ ≥ 30 mg hr/L	↑ LN response w/ MPA level ≥30; *P* = 0.057 AEs: infection (12%), cytopenia (6%) but no correlation w/ levels Weak but significant linear correlation b/w MPA level albumin as well as wt. (R^2^ = 0.222, *P* = 0.017; R^2^ = 0.129, *P* = 0.037)	Target MPA AUC_0–12_ ≥30 mg hr/L promises ↑ LN response
Chariyavilaskul et al (2022)^46^, ^c^	1,440 mg/d	C_0.5_ ≥ 2.03 μg/mL or AUC_0–12_ ≥ 45 μg hr/mL	MPA AUC_0–12_ ≥45 μg h/mL associated w/ better LN response MPA C_0.5_ 2.03 μg/mL could be used as a surrogate for AUC_0–12_ ≥45 μg h/mL (sensitivity = 85%; specificity = 88.2%) Linear correlation b/w UPCR (R^2^ = 0.28), serum albumin, and MPA levels (R^2^ = 0.019)	MPA C_0.5_ ≥2.03 μg/mL could be a surrogate for AUC_0–12_ levels and improves renal response rate in LN
Kittanamongkolchai et al (2013)^38^, ^c^	1.5 g/d	C_1_ > 13 mg/L or AUC_0–12_ > 45 mg hr/L	89% of pts. w/ LN achieved renal remission w/ MPA C_1_ levels >13 mg/L maintained over 24 weeks AEs: mild infection in four pts. w/ MPA C_1_ levels 9–18 (*P* = NS)	Maintaining MPA C_1_ levels >13 mg/L ensures LN remission w/o risking AEs
Mizaki et al (2023)^42^, ^c^	1,500 mg/d	C_max_ < 24.36 μg/mL or AUC_0–12_ 30–45 μg h/mL	No clinical outcomes reported Median MPA C_max_ and MPA AUC_0–12_ in pts. w/ GI AEs (26.3 and 63.4) vs w/o GI AEs (12.3 and 45.3); *P* < 0.05	Maintain C_max_ levels <24.36 μg/mL to reduce AEs
Fujinaga et al (2008)^40^, ^c^	405 mg/m^2^ BID	C_0_ 2–5 mcg/mL	After maintaining MPA C_0_ levels b/w 2–5 for 6–41 months, no LN flares or AE were observed	MPA C_0_ levels lie w/in 2–5 mcg/mL ↑ safety & efficacy
Zabotti et al (2014)^44^, ^c^	3 g/d	AUC_0–12h_ 45–60 mg hr/L	w/ maintaining MPA AUC_0–12_ b/w 45–60; 100% of pts. achieved LN response w/o flares Two pts. had mild transient AEs	Therapeutic range for MPA AUC_0–12_ 45–60 mg hr/L
Lertdumrongluk et al (2010)^35^, ^c^	1,416 mg/d	AUC_0–12_ > 45 mg hr/L	Mean MPA level in responders vs nonresponders w/ LN (65.98 ± 23.77 vs 32.08 ± 7.97); *P* = 0.002 AEs did not correlate w/ MPA levels	Maintaining MPA AUC_0–12_ >45 mg hr/L ↑ LN disease control
Abd Rahman et al (2015)^45^, ^c^	1,500 mg/d	AUC_0–12_ > 30 mg l^−1^	Median AUC_0–12_ level in responders vs nonresponders (23.2 vs 19.2); *P* = 0.103 C_max_ in responders vs nonresponders w/ LN (11.9 vs 6.1); *P* = 0.016 44% of pts. had anemia w/ median AUC_0–12_ level 37.8 mg I^−1^; *P* = 0.038	MPA AUC_0–12_ levels >30 mg l^−1^ increases renal response but risk cytopenia

* AE, adverse event; AKI, Acute Kidney Injury; AUC_0–12_, area under the curve at 0 to 12 hours; BID, twice a day dosing; BILAG, British Isles Lupus Assessment Group; b/w, between; C_12_, concentration at 12 hours; CI, confidence interval; C_max_, maximum mycophenolic acid concentration among serial measurements; CrCl, creatinine clearance; C_trough_, trough concentration; DM, Diabetes Mellitus; dsDNA, double‐stranded DNA; G_6_, mycophenolic Acid Glucuronide 6 hours levels; GFR, glomerular filtration rate; GI, gastrointestinal; LLDAS, Lupus Low Disease Activity State; LN, lupus nephritis; MMF, mycophenolate mofetil; MPA, mycophenolic acid; N/A, not applicable; NPV, Negative Predictive Value 3; NS, not significant; OR, odds ratio; PNA, pneumonia; pt., patient; SLE, systemic lupus erythematosus; SLEDAI, Systemic Lupus Erythematosus Disease Activity Index; TDM, therapeutic drug level monitoring; UPCR, urine protein creatinine ratio; w/, with; wt., weight; w/o, without.

^a^ Outcomes are per SLE outcomes analysis (SLEDAI, BILAG, and LLDAS).

^b^ Outcomes are per serology analysis.

^c^ Outcomes are per LN outcomes analysis (Kidney Disease Improving Global Outcomes and BILAG).

### Association between MPA levels and overall (extrarenal and renal) SLE outcomes

A total of 15 studies examined the association between MPA levels and overall SLE outcomes (renal and extrarenal; Table 2).^6^, ^26^, ^27^, ^30^, ^31^, ^32^, ^34^, ^35^, ^36^, ^39^, ^41^, ^43^, ^45^, ^46^, ^47^ We performed two pooled analyses. The first analysis showed a weight mean difference of 32 in MPA levels in responders compared to nonresponders (95% CI 17.35–45.67; *P* ≤ 0.0001; I^2^ = 81%; Supplementary Figure 2).^6^, ^26^, ^27^, ^32^, ^34^, ^35^, ^36^, ^39^, ^41^, ^43^, ^45^, ^47^ The second analysis estimated 12.2‐fold higher odds of overall SLE response (renal response and extrarenal response) by higher or therapeutic MPA levels including data from seven studies (95% CI 5.44–27.35; *P* < 0.0001; I^2^ = 41%; Supplementary Figure 3).^6^, ^26^, ^30^, ^31^, ^34^, ^43^, ^46^ Certainty of evidence was rated as very low for overall SLE outcomes by GRADE, primarily due to imprecision and study limitations. Narrative synthesis of one study revealed that MPA C_trough_ (concentration at zero hours [C_0_]) between 2 and 5 μg/mL were not associated with reduced LN flare frequency over 6 to 41 months.^40^

### Association between MPA levels and adverse events

**Figure 4:**
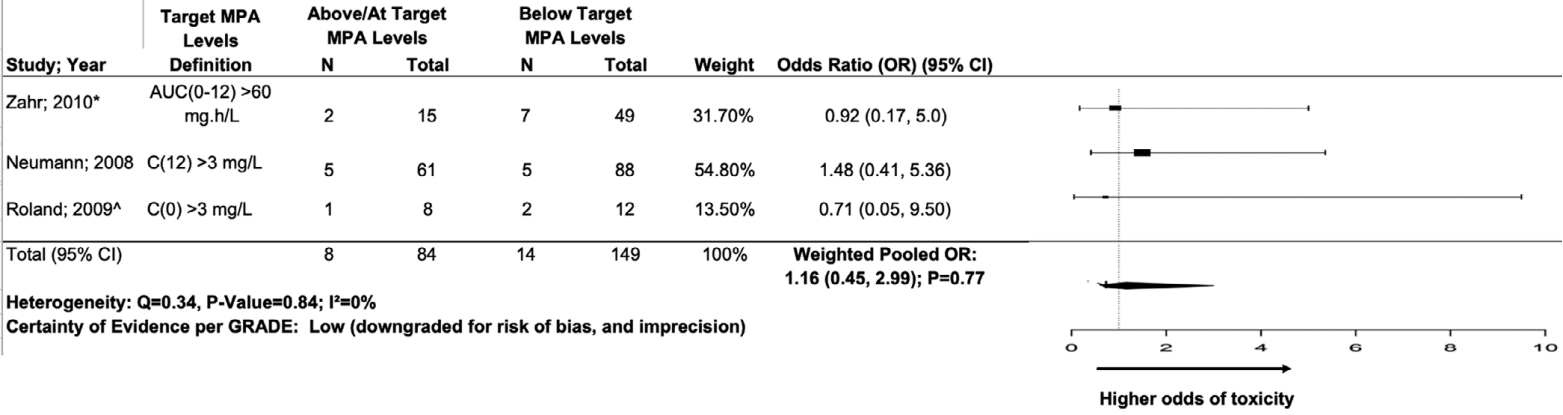
Forest plot showing odds of adverse events/toxicity in patients with systemic lupus erythematosus with extrarenal manifestations by target or higher mycophenolic acid (MPA) levels. AUC(0–12), area under the curve at 0 to 12 hours; C(12), concentration at 12 hours; CI, confidence interval; GRADE, Grading of Recommendations Assessment, Development, and Evaluation; OR, odds ratio. *Zahr et al^6^ excludes patients with low levels and cytopenia due to active lupus; ^C(0) was used for safety analysis given strong R association between C(0) and AUC(0–4) per study.

Three studies examined associations between MPA levels and adverse events in patients with extrarenal SLE (Table 2).^6^, ^31^, ^36^ Our pooled analysis did not demonstrate a statistically significant increase in toxicity for threshold of 60 mg hr/L AUC_0–12_ and ≥3 mg/L MPA C_trough_ (pooled OR 1.16, 95% CI 0.45–2.99; *P* = 0.77; I^2^ = 0%; Figure 4). Certainty of evidence was rated as very low for adverse events by GRADE, primarily due to imprecision and study limitations. Narrative synthesis of data revealed that the most frequently cited adverse events were gastrointestinal symptoms, followed by cytopenia and infections. Five studies reported no associations between MPA levels and adverse events.^6^, ^34^, ^35^, ^37^, ^40^ One noted higher median MPA maximum MPA concentration among serial measurements (C_max_) and AUC_0–12_ levels in patients experiencing gastrointestinal side effects (median MPA C_max_ in patients with vs without adverse events 26.3 vs 12.3 μg/mL) versus (median MPA AUC_0–12_ in those with vs without adverse events 63.4 vs 45.3 μg h/mL).^42^ Another study reported that 37.5% of patients developed mild adverse events at a range of MPA AUC_0–12_ 60 to 90 mg hr/L.^34^ Meanwhile, a study including patients from Southeast Asia noted a significant correlation between cytopenia and higher MPA levels (median AUC_0–12_ 37.8 mg I^−1^; *P* = 0.038).^45^

### Sensitivity analysis and publication bias

In sensitivity analyses restricted to prospective or retrospective cohorts, therapeutic MPA levels remained consistently associated with greater odds of clinical response, supporting the robustness of our findings. Visual inspection of funnel plots (Supplementary Figures 4–9) suggests possible publication bias across analyses.

### Association between MPA levels and clinical variables

Four examined clinical variables that might affect MPA levels included serum albumin (n = 9), weight (n = 3), eGFR (n = 3), and concomitant steroid use (n = 2; Tables 1 and 2). A linear correlation between MPA levels and serum albumin was noted in four studies; two studies reported a weak correlation (R^2^ = 0.22 and R^2^ = 0.28; *P* = 0.017 and 0.002); five studies noted no correlation between albumin and MPA levels.^6^, ^26^, ^30^, ^36^, ^37^, ^41^, ^43^, ^46^, ^47^ Additionally, MPA levels did not correlate with weight in two studies (one study observed a weak linear correlation between MPA levels and dose per body weight, R^2^ = 0.129; *P* = 0.037).^26^, ^36^, ^37^ One study found a significant association between MPA levels and prednisone use (strength of association not reported).^43^ Direct correlation between MPA levels and adherence was not measured. One study noted single‐nucleotide polymorphism (SNP) rs2273697 A/G genotype was linked with lower MPA exposure, warranting higher MPA dosing.^28^ One study reported a need to increase the MMF dose from 1 to 2 g/day to achieve a target MPA AUC_0–12_ range of 30 to 60 mg hr/L.^37^

### Measuring MPA levels and therapeutic threshold or therapeutic range for MPA levels

MPA levels were measured using liquid chromatography in 9 studies,^6^, ^26^, ^30^, ^33^, ^34^, ^37^, ^41^, ^44^, ^46^ both mass spectrometry and liquid chromatography in 4 studies,^32^, ^39^, ^45^, ^47^ and enzyme assays in 10 studies,^27^, ^28^, ^31^, ^35^, ^36^, ^38^, ^40^, ^42^, ^43^, ^48^ and 1 study did not specify their measurement technique.^29^ MPA levels were measured in plasma in 18 studies^26,27,29–35,38–41,43–47^ and serum in 6 studies.^6^, ^28^, ^36^, ^37^, ^42^, ^48^

Studies used different methods of measuring MPA. To elaborate, AUC MPA levels estimated MPA levels over a time period to obtain a dose: time AUC MPA concentration (AUC time period in hours) was the most common measurement method. A total of 13 studies reported AUC_0–12_, 1 study reported AUC_0–24_, whereas one study measured AUC_0–4_ and AUC_0–3_.^6,26,27,30,33–38,42–46^ Two studies reported and used the C_max_.^42^, ^45^ Other studies measured MPA predose concentration (C_0_).^36^, ^39^, ^40^ Time‐dependent assays measured a single MPA level at 1 hour after MPA dose (C_1_), 0.5 hours after the last dose (C_0.5_), and 6 and 12 hours from the last dose (C_6_ and C_12_).^28^, ^29^, ^31^, ^38^, ^46^, ^47^

Therapeutic thresholds or therapeutic ranges for MPA levels varied across studies. The most used therapeutic threshold was MPA AUC_0–12_ >30 mg hr/L,^33^, ^37^ and a reference range of 30 to 60 mg hr/L was commonly used. Two studies used a therapeutic range of MPA AUC_0–12_ 45 to 60 mg hr/L^42^ and 60 to 90 mg hr/L.^34^ Studies reporting a single MPA predose concentration (C_12_) commonly used therapeutic ranges between 1 and 2.5 mg/L (Tables 1 and 2). Therapeutic ranges and thresholds for MPA AUC_0–12_ and C_0.5–1_ levels are shown in Tables 1 and 2.

## DISCUSSION

MPA is a key therapeutic in the SLE treatment armamentarium, yet clinical effectiveness and safety of MPA are heterogeneous due to variability in absorption, metabolism, drug interactions, and other clinical factors.^8^ TDM of MPA could pose a solution for this conundrum by guiding safe and effective dosing of MPA. Our study emphasizes the clinical significance of monitoring MPA levels in all patients with SLE, including extrarenal and renal manifestations. This meta‐analysis noted 12‐fold higher odds of overall SLE response in patients with an MPA level cutoff ≥30 to 35 mg hr/L AUC_0–12_ or ≥1.5 mg/L C_trough_. In patients with extrarenal manifestations, this MPA threshold was associated with significantly higher 15‐fold odds of clinical response as well as a seven‐point reduction in SLEDAI score (95% CI 2.7–11). Additionally, we found MPA levels were 32 units higher (95% CI 17.35–45.67) in overall responders and 39 units (95% CI 15.05–62.53) higher in patients with extrarenal SLE manifestations. Our pooled analysis of adverse events did not demonstrate a statistically significant increase in toxicity at higher exposures (MPA AUC_0–12_ 60 mg hr/L and C_trough_ ≥2.5–3 mg/L). Synthesizing these findings, the evidence supports maintaining MPA levels ≥30–35 mg hr/L AUC_0–12_ or 1.5–2.5 mg/L C_12_ trough is associated with better clinical response. Although data suggest that adverse events may emerge at MPA levels beyond 60 mg hr/L MPA AUC_0–12_ and ≥2.5 to 3 mg/L C_trough_. The findings of this meta‐analysis and systematic review underscore the importance of TDM for MPA during routine SLE visits including both renal and extrarenal manifestations and could guide MPA dosing, balancing safety versus efficacy in SLE. Additionally, higher‐quality longitudinal studies are needed to confirm these findings and inform a workflow for clinical adoption and to guide MPA dosing in SLE.

The 2023 update from EULAR^14^ recommends MPA as one of the key therapeutics for management of SLE. Specifically, in extrarenal SLE, the European Renal Association guidelines recommended MPA as first‐line therapy for moderately active systemic lupus (SLEDAI > 6).^14^ Additionally, the 2024 ACR guidelines recommends mycophenolate as the preferred backbone therapy in the triple‐regimen recommendation for patients with class III/IV LN with significant proteinuria.^50^ However, there is variability in plasma concentrations of MPA, resulting in heterogeneity in clinical response. Moreover, arbitrarily increasing MPA doses can result in toxicity, particularly in groups who are at higher risk of side effects from MPA. Thus, titration of MPA dose based on individual patient factors is needed to balance efficacy and safety in clinical practice.^8^, ^36^, ^37^ TDM can streamline titration of MPA dose, which is widely used in transplant and allows for personalizing MPA dose and reduces the risk of treatment failure by 39%.^51^ Likewise, in LN, a recent meta‐analysis reported 3.2‐fold higher odds (95% CI 1.2–8.4) of renal response with therapeutic MPA levels (AUC_0–12_ 30–60 mg hr/L).^13^ Similar data are lacking for extrarenal manifestations of SLE—more than 50% of patients with SLE. In the absence of such data, patients with extrarenal SLE manifestations often receive suboptimal dosing, thus increasing the risk of flares or adverse events. Supporting this, Woillard et al and Zahr et al both reported low or subtherapeutic MPA plasma levels with standard or fixed MPA dosing resulting in higher lupus flares or active lupus (SLEDAI > 6).^6^, ^30^ Our meta‐analysis highlights the potential impact of TDM in SLE by reporting 15‐fold higher pooled odds (95% CI 4.74–46.89) of clinical response with higher MPA levels. Across individual studies, efficacy was most consistently observed at exposures above AUC_0–12_ >30 to 35 mg hr/L or MPA C_12_ trough >1.5 mg/L, particularly in patients with SLE with extrarenal manifestations. Higher MPA exposures were also associated with a seven‐point reduction in SLE disease activity (95% CI 2.74–19.99); extrarenal responders had 39 mg hr/L higher MPA levels compared to nonresponders (95% CI 15.05–62.53).

Upon summarizing evidence on toxicity, we noted no significant associations between higher cutoff/threshold of 60 mg hr/L AUC_0–12_ and ≥3 mg/L MPA C_trough_ (pooled OR 1.16, 95% CI 0.45–2.99; *P* = 0.77). Similar findings were noted in a recent study in which the weight mean difference (5.1, 95% CI −5.2 to 15.4) in MPA levels did not differ in patients with versus without adverse events.^13^ Although pooled evidence revealed no statistically significant associations, two studies reported an inverse relationship with cytopenia (anemia) and infections with higher median MPA AUC_0–12_ levels (37.8 mg I^−1^)^45^ and higher MPA C_12_ levels (4.3 mg/L).^28^ Side effects or adverse events reported in both studies were mild and reversible. No life‐threatening events were reported with higher MPA levels. These studies also noted that adverse events could depend on clinical factors such as prednisone dose, Ig level, or variability in drug metabolism due to genotypic variations (SNP rs2273697 A/G genotype).^28^ Certainty was rated as low across studies. These ratings highlight that although associations were consistent, the strength of evidence was limited and underscored a need for higher‐quality longitudinal studies.

A final question that needs to be answered to empower clinicians to use MPA level monitoring in SLE visits is how and when to measure MPA levels. Target levels and reporting methods varied among the studies; however, MPA AUC_0–12_ was the most frequently used, and a therapeutic threshold of >30 mg hr/L was recommended by several studies for clinical use. One study noted better response rates with MPA AUC_0–12_ levels in the 60 to 90 mg hr/L range.^34^ The feasibility of using MPA AUC_0–12_ in clinics is questionable given that calculating an AUC_0–12_ requires multiple samples at different intervals following MPA administration. Alternatively, a 12‐hour trough MPA level might be easier to reproduce and was the second most common method used.^36^, ^39^, ^40^, ^43^ It was noted that maintaining MPA C_12_ levels at or above 1.5 mg/L was associated with fewer flares and better clinical response. Future studies on the clinical significance and reliability of maintaining levels within the aforementioned therapeutic ranges are needed.

Despite strengths of this study including analyzing the use of TDM of MPA to improve SLE treatment efficacy and balance safety, particularly in patients with extrarenal manifestations, we acknowledge our limitations. First, most studies were conducted in Europe or Asia; only two studies were done in the United States.^29^, ^33^ This could impact generalizability of the summarized evidence. Second, high heterogeneity (I^2^ 90%) was observed among studies. However, heterogeneity reduced after including only patients with extrarenal manifestations. To further address heterogeneity, we performed sensitivity analysis restricted to prospective or retrospective cohort studies. These confirmed that therapeutic MPA levels were consistently associated with greater odds of clinical response, supporting the robustness of our findings despite variation in study design. Third, most studies used the MPA AUC_0–12_ reporting method; however, real‐world clinical feasibility could be limited. MPA C_12_ levels could be feasible, but only three studies measured it. Fourth, only a limited number of studies examined the longitudinal impact and feasibility of using MPA levels in clinics; thus, further data are needed on when and how frequently MPA levels should be monitored during SLE visits. Fifth, the absence of patient‐reported outcomes measures in the included studies limits the ability to evaluate the patient perspective on treatment benefit. Finally, as reflected in our GRADE assessment, the certainty of evidence was very low across outcomes, further emphasizing the need for additional longitudinal studies.

In conclusion, in this systemic review and meta‐analysis, high or therapeutic MPA levels were associated with 15‐fold higher pooled odds of clinical response in patients with extrarenal SLE and 12‐fold higher odds of overall response in extrarenal and renal outcomes. Additionally, a clinically significant seven‐point reduction in SLE disease activity was noted with higher MPA levels. Across individual studies and pooled analysis, efficacy was most consistently observed at MPA AUC_0–12_ ≥30–35 mg hr/L or C_12_ trough 1.5 to 2.5 mg/L. MPA AUC_0–12_ was a frequently used method; however, 12‐hour trough MPA levels might be easier to adopt in nonresearch clinical settings. No statistically significant association between adverse events and higher thresholds was noted; however, several studies suggest that adverse events may emerge at exposures beyond 60 mg hr/L MPA AUC_0–12_ and ≥2.5 to 3 mg/L C_trough_. Future research should focus on defining a therapeutic range for MPA level that balances safety and efficacy and examining the longitudinal impact of TDM of MPA in SLE. These findings support the clinical utility of TDM to guide precise MPA dosing to enhance efficacy in all patients with SLE, including both extrarenal and renal manifestations. However, ideal testing method, timing of testing, cost‐effectiveness, and how to incorporate discussions on TDM to enhance shared decision‐making during SLE visits need to be further studied.

## AUTHOR CONTRIBUTIONS

All authors contributed to at least one of the following manuscript preparation roles: conceptualization AND/OR methodology, software, investigation, formal analysis, data curation, visualization, and validation AND drafting or reviewing/editing the final draft. As corresponding author, Dr Garg confirms that all authors have provided the final approval of the version to be published, and takes responsibility for the affirmations regarding article submission (eg, not under consideration by another journal), the integrity of the data presented, and the statements regarding compliance with institutional review board/Declaration of Helsinki requirements. All authors contributed to the investigation, formal analysis of the data, and writing, reviewing, and editing of the manuscript.

## Supporting information


**Disclosure form**.

**Supplementary Table 1:** A Priori Inclusion and Exclusion Criteria
**Supplementary Figure 1:** PRIMSA Flow Diagram Showing Literature Screening Process
**Supplementary Figure 2:** Forest plot showing weighted mean difference in MPA levels by overall SLE response in patients with renal and extra‐renal manifestations
**Supplementary Figure 3:** Forest plot showing the odds of overall clinical response in SLE patients with extra‐renal and renal manifestations by MPA levels
**Supplementary Figure 4:** Funnel plot for pooled odds of clinical response in SLE patients with extra‐renal manifestations by target MPA levels
**Supplementary Figure 5:** Funnel plot for weighted mean difference in MPA levels by clinical response in SLE patients with extra‐renal manifestations
**Supplementary Figure 6:** Funnel plot for the weighted mean difference in SLE disease activity by higher or therapeutic MPA levels in SLE patients with extra‐renal manifestations
**Supplementary Figure 7:** Funnel plot for weighted mean difference in MPA levels by overall SLE response in patients with renal and extra‐renal manifestations
**Supplementary Figure 8:** Funnel plot for odds of overall clinical response in SLE patients with extra‐renal and renal manifestations by MPA levels
**Supplementary figure 9:** Funnel plot for odds of adverse events/toxicity in SLE patients with extra‐renal manifestations by target or higher MPA levels
