# Supplementary material for: Clinical Significance of Therapeutic Drug Level Monitoring for Mycophenolate in Patients With Extrarenal Systemic Lupus Erythematosus—A Systematic Review and Meta‐Analysis
Source: Arthritis Care Res (Hoboken). 2026 Apr 22;78(10):1424–37. doi: 10.1002/acr.80035 (PMC13499014; doi:10.1002/acr.80035)
Supplement: Supplementary file 2 [file ACR-78-1424-s002.docx]

Supplementary Table 1. A Priori Inclusion and Exclusion Criteria

Inclusion criteria

1. Studies including patients with systemic lupus erythematosus (SLE or lupus) taking MMF
2. Studies including human subjects
3. Studies measuring MMF or MPA levels
4. Full text available upon request or online
5. Studies examining an association between MMF/MPA levels and adherence OR disease activity OR treatment response (e.g., CKD progression; change in LN disease activity; change in SLE disease activity defined as improvement in standard SLE disease score or change in prednisone dose; change in flare (renal or extra-renal) frequency; change in SLE response defined by the primary study) OR toxicity or adverse events (including severe or non-severe events as defined by the primary study)
6. Any disease manifestation of SLE was included

Exclusion criteria

1. Animal studies
2. Studies including patients with a kidney/organ transplant
3. Studies done on other autoimmune disease (non-SLE)
4. Studies not measuring MMF/MPA levels or including data on SLE outcomes or adverse events/toxicity
5. Gray literature including conference abstracts, case reports, conference proceedings, reviews, expert opinions

**Supplementary Figure 1. PRIMSA Flow Diagram Showing Literature Screening Process**

**Supplementary Figure 2. Forest plot showing weighted mean difference in MPA levels by overall SLE response in patients with renal and extra-renal manifestations**

**Supplementary Figure 3. Forest plot showing the odds of overall clinical response in SLE patients with extra-renal and renal manifestations by MPA levels**

**Supplementary Figure 4. Funnel plot for pooled odds of clinical response in SLE patients with extra-renal manifestations by target MPA levels**

**Supplementary Figure 5. Funnel plot for weighted mean difference in MPA levels by clinical response in SLE patients with extra-renal manifestations**

**Supplementary Figure 6. Funnel plot for the weighted mean difference in SLE disease activity by higher or therapeutic MPA levels in SLE patients with extra-renal manifestations**

**Supplementary Figure 7. Funnel plot for weighted mean difference in MPA levels by overall SLE response in patients with renal and extra-renal manifestations**

**Supplementary Figure 8. Funnel plot for odds of overall clinical response in SLE patients with extra-renal and renal manifestations by MPA levels**

**Supplementary figure 9. Funnel plot for odds of adverse events/toxicity in SLE patients with extra-renal manifestations by target or higher MPA levels**
